# Postoperative Open Reduction Internal Fixation Discomfort and Rehabilitation Response: A Case Report

**DOI:** 10.7759/cureus.90031

**Published:** 2025-08-13

**Authors:** Evan C Xu, Asante Hohn

**Affiliations:** 1 Outpatient Physical Therapy Clinic, Attain Physical Therapy, Berkeley Heights, USA

**Keywords:** lower extremity functional scale, manual muscle testing, open reduction–internal fixation, patellar fracture, physical therapy, postoperative rehabilitation

## Abstract

Rehabilitation details after open, comminuted patellar fractures in older adults are seldom published; this case maps a full 20-week recovery using a structured, criteria-based program. A healthy 56-year-old woman was unable to bear weight after a fall, with anterior knee wounds and imaging that showed an open, comminuted left-patella fracture. Six weeks postoperatively, she reported resting pain of 4/5 on the Verbal Rating Scale (VRS) and demonstrated only 40° active range of motion (AROM). The injury was classified as a Type I/II open patellar fracture. It was stabilized with open reduction and internal fixation (ORIF) with tension-band wiring plus screws. Beginning at postoperative Week 6, the patient followed a five-phase outpatient protocol progressing from immobilized isometrics to closed kinetic chain (CKC) functional drills over ≈42 visits. By Week 20, active flexion increased to 129°, quadriceps strength to 4+/5, and the Lower Extremity Functional Scale (LEFS) score to 50/80. Mid-patella circumference fell 1.8 cm, serial radiographs confirmed union, and resting pain fell to 2/5, although intermittent stiffness and mild activity-related aching persisted. A staged, adaptable rehabilitation plan can yield substantial gains in mobility, strength, and function within five months of an ORIF procedure for complex patellar fractures. Daily low-load motion and progressive closed-chain loading appear key, and patients should be advised that modest pain and strength asymmetry may linger beyond 20 weeks, warranting continued follow-up.

## Introduction

Patellar fractures account for about 1% of all skeletal injuries and show a pronounced age- and sex-related rise, climbing from 15.4 per 100,000 in males from 10 to 19 years of age to approximately 36 per 100,000 in women from 60 to 80 years of age, the highest incidence [1]. During the past decade, from 2006 to 2020, the number of patellar surgical interventions has increased from 50% in 2006 to 75% in 2020, with the majority of surgical interventions occurring in individuals more than 50 years of age [2]. Open reduction and internal fixation (ORIF) is regarded as the preferred technique for displaced or comminuted patterns, yet existing literature rarely details the postoperative rehabilitation sequence or functional milestones, especially when an open fracture and early infection risk complicate care and intervention. Additionally, there is a large focus on hardware complications, as 22.53% of patients proceeded to suffer from hardware complications that this single-patient case report does not cover [3]. 

We describe a healthy 56-year-old woman who sustained an open, comminuted left-patellar fracture with intra-articular air requiring urgent irrigation-debridement followed by tension-band ORIF. What distinguishes this report is the prospectively recorded, five-phase outpatient program that linked objective gains in range of motion, strength, limb girth, and patient-reported function to pre-specified loading criteria across the first 20 weeks. Additionally, pain was monitored with the verbal rating scale (VRS) established by expert opinion, where 0/5 = no pain, 1/5 = very mild, 2/5 = mild, 3/5 = moderate, 4/5 = severe, 5/5 = very severe. Pain response from the patient structured the rehabilitation program via limiting/progressing weight bearing and additional exercises for recovery and strengthening. By integrating surgical details with day-to-day physiotherapy decisions, this case provides granular, clinically actionable data that complements existing series focused mainly on radiographic union or final knee flexion.

## Case presentation

Patient information

A 56-year-old woman with no reported comorbidities, regular medications, or history of knee pathology sustained an open, comminuted fracture of her left patella after a mechanical fall. Her primary concern was ambulating and carrying out day-to-day activities with this injury, with the major symptoms following the operation being the limited range of motion in the knee. She denied tobacco and alcohol use, and there is no record of relevant family, medical, or psychosocial factors. There are no past interventions and outcomes that are relevant to her traumatic injury. 

Clinical findings

On arrival to the emergency department, the patient was unable to bear weight, the left patella appeared dislocated anteriorly, and several small lacerations were noted over the anterior knee. Distal pulses and sensation were intact. Pain was rated 5/5 VRS, swelling was moderate, and active motion was absent. Six weeks after surgery, at her physical-therapy initial evaluation, the patient demonstrated 40° active and 45° passive knee flexion, quadriceps and hamstring strength of 2-/5 by manual muscle testing (MMT), marked edema (left mid-patella circumference = 40 cm versus 38 cm on the right), and a lower extremity functional scale (LEFS) score of 11/80. She reported constant aching pain and an inability to bear full weight.

Timeline

Relevant information, time from injury, approximated postoperative week, and specific details are provided as a general overview of the events that occurred during postoperative care. Updates are provided by physical therapy rehabilitation. The following table describes the program the patient followed (Table 1).

**Table 1:** Chronological timeline of surgery and rehabilitation milestones after left patellar open reduction internal fixation procedure. ORIF: Open reduction-internal fixation; WBAT: Weight bearing as tolerated; AROM: Active range of motion; PROM: Passive range of motion; LEFS: Lower extremity functional scale; ROM: Range of motion

Time from Injury	Approximated Postoperative Week	Event
Day 0	–	Mechanical fall → Emergency department presentation• Plain radiograph and computed tomography confirm open, comminuted left-patellar fracture• Knee immobiliser applied
Day 1	–	Urgent irrigation–debridement and ORIF (tension-band wire + screws)• Post-operative radiograph: anatomic reduction
Day 3	–	Discharged home with hinged knee brace (locked 0–30°); weight bearing as tolerated (WBAT) in extension
Week 6	0; Rehabilitation Begins	Physical-therapy intake (Visit 1): 40° Active range of motion (AROM) / 45° passive range of motion (PROM) flexion; quadriceps 2-/5; Lower extremity functional scale (LEFS) 11/80
Week 10	≈ 4	Visit 11: brace unlocked at night; flexion 85° AROM; quadriceps 2+/5; LEFS 20/80
Week 14	≈ 8	Visit 24: full range of motion (ROM) target phase begins; flexion 113° AROM; quadriceps 3+/5; LEFS 32/80
Week 17	≈ 11	Visit 34: brace discontinued; flexion 123° AROM; quadriceps 4-/5; LEFS 43/80
Week 20	≈ 14	Visit 42: radiographic union confirmed; flexion 129° AROM / 136° PROM; quadriceps 4+/5; LEFS 50/80

Diagnostic assessment

**Figure 1:**
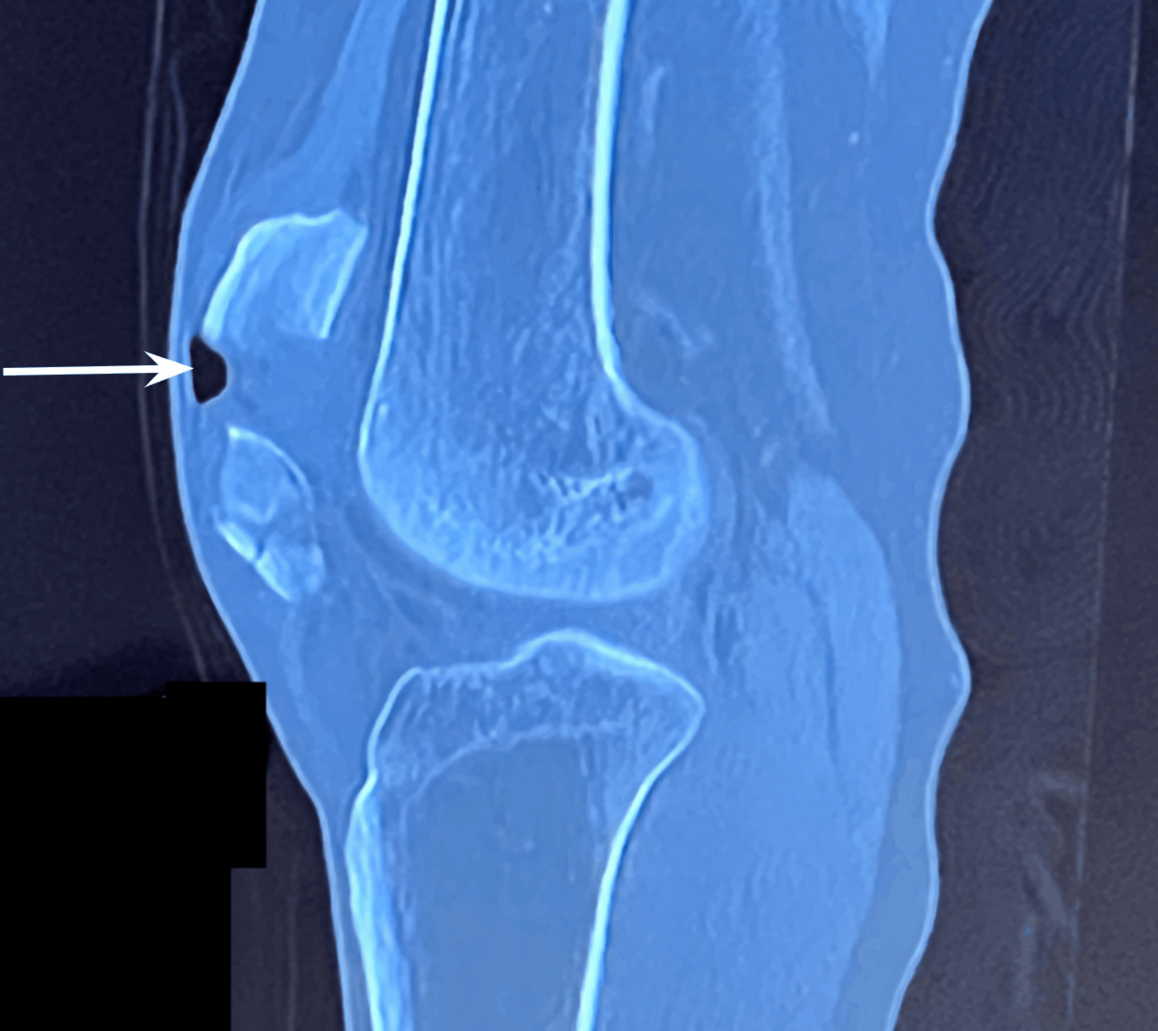
Pre-operative sagittal computed tomography scan of the left knee indicating multi-fragment, displaced patellar fracture, and intra-articular air revealing an open fracture.

Plain radiographs obtained in the emergency department demonstrated a multi-fragment, displaced patellar fracture. A same-day computed-tomography scan confirmed comminution and revealed intra-articular air, establishing the fracture as open. Imaging on the day in the emergency department is shown below, which demonstrates a comminuted fracture of the patella with a small focus of intra-articular air, indicated by a white arrowhead, reflecting the open nature of the injury (Figure 1).

A postoperative radiograph on day one showed an anatomic reduction secured with tension-band wiring and cannulated screws. An additional radiograph taken approximately 12 weeks postoperatively demonstrated bridging cortex and trabecular continuity and was read as radiographic union. No laboratory values or culture results were reported.

Surgical management

Around 24 hours after injury, the patient underwent urgent irrigation and debridement followed by open reduction and internal fixation (tension-band wire plus screws). Pre-operative prophylaxis consisted of intravenous cefazolin. A hinged knee immobiliser locked at 0-30° was applied postoperatively, and the patient was discharged on postoperative Day 3 with instructions to bear weight as tolerated in full extension. The following implants were used in the surgical open reduction internal fixation procedure (Table 2).

**Table 2:** Hardware implanted during open reduction internal fixation of the left patella, indicating level of intervention critical for future analysis. All implants used were manufactured by Arthrex (Arthrex Inc., Naples, FL).

Implants	LRB	No. Used	Action
Patella Suture Plate Small	Left	1	Implanted
Arthrex Patella Plate	Left	1	Implanted
Arthrex 3.0 Washer	Left	1	Implanted
Scrw Cort Lo Prof 3.0x18mm-	Left	1	Implanted
Arthrex 3.0x16 Locking Screw	Left	1	Implanted
Arthrex 3.0x14 Locking Screw	Left	2	Implanted
Arthrex 3.0x18 Locking Screw	Left	1	Implanted
Arthrex 3.0x40 Cannulated Screw	Left	1	Implanted

**Figure 2:**
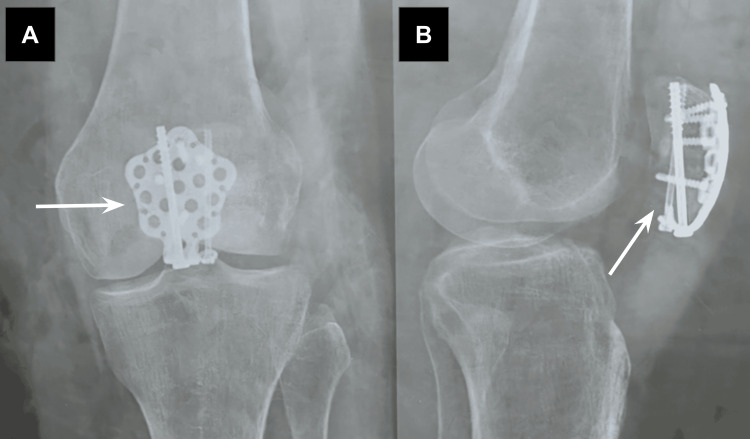
Left knee—anteroposterior (A) and lateral radiographs (B) indicating the result of the open reduction internal fixation procedure. Radiographs were obtained on postoperative day 1.

Immediately following the surgery on postoperative Day 1, the patient received an anteroposterior radiograph and a lateral radiograph of her left knee in order to assess the success and outcome of the open reduction internal fixation procedure. The radiograph is shown below, with implants indicated by white arrowheads (Figure 2).

Outpatient rehabilitation

Formal rehabilitation began at postoperative Week 6 and followed a five-phase, criteria-based program. Exercise protocol is established by expert opinion, utilizing initial data collected upon physical therapy intake. Data includes LEFS score, AROM, PROM, and surgical history. Pain, measured by VRS, was also used to help structure the rehabilitation program by limiting or progressing certain activities for maximum physical rehabilitation without risk of injury or worsening pain.

In phase one (rehabilitation Weeks 0-6), the knee brace was locked at 0-30°. Treatment emphasized isometric quadriceps and hamstring sets, straight-leg raises, PROM, and pain-modulating modalities.

Phase two (Weeks 6-10) permitted the removal of the brace at night; knee flexion was advanced by 15° each week until 90° was reached. Exercises included heel slides, terminal knee extensions (TKE) with an elastic band, and progressive ankle-carrying activities.

In phase three (Weeks 10-12), the brace was unlocked for gait. Full range of motion was targeted, and eccentric quadriceps control was introduced with yoga-ball-assisted TKEs and light resistance bands.

In phase four (Weeks 14-16), the brace was discontinued. Closed-chain strengthening and proprioceptive drills were added, including half-crank cycling, banded side steps, wall sits, and single-leg bridges.

Phase five (Week 16 onward) marked the return to unrestricted daily activities. Higher-load closed-chain tasks such as Spanish squats, sled pushes, and step-downs were prescribed to restore power and endurance.

Each visit began with 28-30 minutes of retrograde massage and PROM, followed by an exercise circuit tailored to the patient’s tolerance. Volume and resistance progressed when pain at rest was ≤2/5 VRS, and swelling remained stable.

Follow-up and outcomes

**Figure 3:**
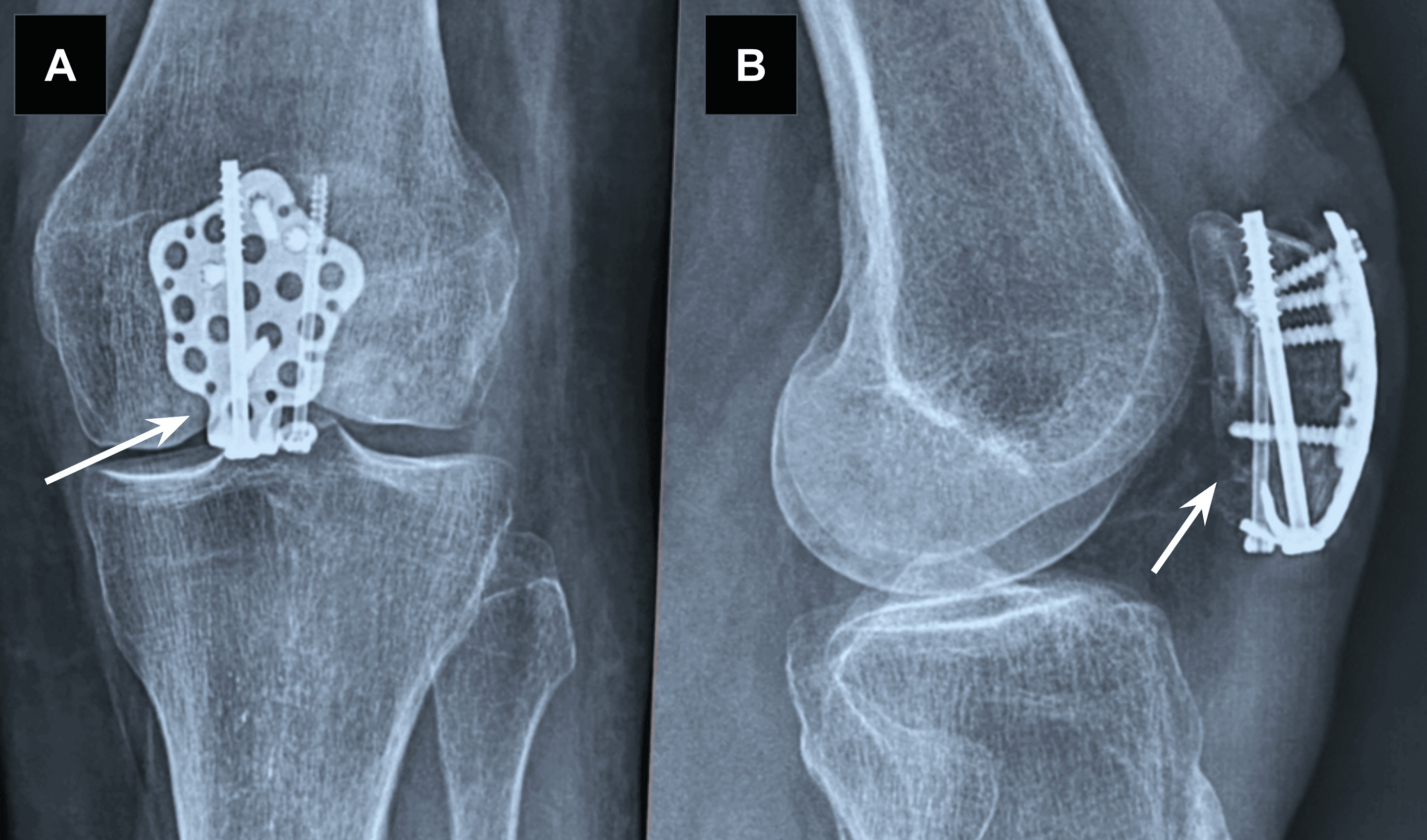
Left knee—anteroposterior (A) and lateral (B) radiographs indicating complete osseous union and no wound complications at postoperative Week 23.

After approximately 20 weeks and 42 outpatient visits, the patient achieved 129° active and 136° passive left-knee flexion, quadriceps strength of 4+/5 and hamstring strength of 4/5, and a reduction in mid-patellar girth of 1.76 cm. LEFS improved from 11 to 50/80, and the patient could ascend stairs reciprocally, ambulate without a brace, and perform light household tasks. Pain ranged from 0/5 VRS at best to 3/5 VRS at worst and was characterized by intermittent aching and stiffness. Radiographs demonstrated complete osseous union, and no wound complications were observed, with the site of union indicated by white arrowheads (Figure 3). Ongoing outpatient therapy was recommended to address residual strength asymmetry and episodic stiffness. No adverse or unanticipated events occurred, and intervention adherence/tolerability was assessed subjectively with MMT and measurements in ROM. The clinician and patient assessed that the bone had completely healed and was discharged from the medical provider as of the 23rd postoperative week, with a recommendation from the clinician to continue physical therapy rehabilitation. 

## Discussion

Clinical course in context

ORIF remains the first-line treatment for displaced or comminuted patellar fractures because it restores and provides rigid internal fixation to the extensor mechanism and permits earlier motion than non-operative care [4]. The patient’s radiographic healing by week 20 (<6 months) and her return of 129° active flexion align with existing studies (Lin 2015 study/subgroup) reporting an average of 127.7° flexion (standard deviation: 16.6) within 12-20 weeks after an ORIF procedure [5]. The patient’s residual strength asymmetry and symptomatic shortcomings (4+/5 vs 5/5) and LEFS score of 50/80 likewise mirror the functional deficits experienced by patients with even 6.5 years (average time of 40 individual patients) of post-operative treatment and recovery [6]. Thus, the patient’s trajectory can be considered typical for a mid-fifties patient with an open, comminuted injury.

Rationale for the staged rehabilitation protocol

In phase one (0-6 weeks), strict immobilization in 0-30° flexion was enforced to limit quadriceps tension across the fixation while permitting protected weight-bearing to minimize osteopenia and enhance cartilage perfusion. Isometric quadriceps sets and straight-leg raises exploit the length-tension curve to maintain motor-unit recruitment without introducing shear at the fracture site.

In phase two (6-10 weeks), gradual 15° weekly flexion gains were designed to stay below the 300 newton (N) peak patellofemoral forces measured at 60-90° during closed-chain tasks [7]. Supine heel slides and TKEs added low-load, high-repetition motion that stimulated synovial fluid distribution and collagen alignment.

In phase three (10-12 weeks), once 90° is painless, unlocked bracing and light resistance (elastic bands, yoga ball TKEs) shifted the emphasis toward eccentric quadriceps control-critical for negotiating stairs and decelerating gait.

Phase four (14-16 weeks), discontinuing the brace allowed for proprioceptive re-education. Half-crank cycling limited compressive load yet provided cyclical motion, which has been shown to enhance nutrient diffusion through hyaline cartilage.

Phase five (>16 weeks) incorporated Spanish squats, sled pushes, and single-leg step-downs to introduce heavier closed-chain loading that mimicked daily functional demands while safely distributing forces across the healing patella.

The selective progression from open-chain to closed-chain activity is supported by EMG studies showing that high load open kinetic chain (OKC) strengthening between 40-90° of motion should be restricted, whereas closed chain kinetic (CKC) strengthening from 0-80° of motion can have comparable quadriceps activation levels with reduced PFJ [8].

Interplay of swelling, scar-mediated stiffness, and pain

Despite radiographic union, the patient reported episodic stiffness and aching. Early cryotherapy and retrograde massage likely mitigated effusion, as evidenced by the 1.76 cm reduction in mid-patella circumference from visit 11 to visit 42; however, intermittent inactivity between sessions may have allowed fibrin cross-linking to reform, recreating the “stiff-sore” cycle she described. This underscores the importance of daily low-load ROM (e.g., stationary cycling) even outside formal therapy.

Strength recovery and functional correlation

Quadriceps MMT improved from 2-/5 to 4 /5, paralleling LEFS gains from 11 → 50. Although MMT is ordinal and ceiling-limited, the consistent stepwise relationship (≈ 1 strength grade ≈ 10-15 LEFS points) reaffirms its clinical utility. Objective isokinetic dynamometry could quantify torque deficits more precisely, but previous work suggests that once MMT reaches 4/5, patients possess ≥60% limb symmetry index, adequate for most activities of daily living (ADL), but below the ≥94.8% threshold recommended for return to sports (RTS) [9]. Therefore, extending therapy to emphasize concentric/eccentric power (plyometrics, resisted cycling) may close the residual gap.

Implications for practice

Individualization Within a Template

While the protocol provided a scaffold, real-time adjustments (e.g., adding band suspension-training straps for balance or downgrading load on high-pain days) were pivotal. This flexible approach suits the heterogeneous healing kinetics seen in older adults or open fractures.

Objective Monitoring 

Incorporating wearable inertial sensors or smartphone-based goniometers could give daily ROM and step-count data, alerting clinicians to plateaus earlier than the current 10-visit interval.

Psychosocial Overlay

The patient’s willingness to “push through” discomfort correlated with larger LEFS jumps after each MD brace-unlock milestone, suggesting that clear goal-setting and visible radiographic milestones bolster adherence.

Future directions

Prospective multi-center studies comparing early-motion vs delayed-motion protocols in open patellar fractures could clarify optimal timing. Additionally, trials of adjuncts such as low-intensity pulsed ultrasound, blood-flow-restriction training, or hydrotherapy may reveal cost-effective accelerants to strength symmetry and pain resolution. The patient may have reached functional goals in angle measurements in her range of motions, but she is still experiencing discomfort and soreness due to immobilization during her post-operative time of six weeks. This is because of scar tissue and adhesion. It can be inferred that the patient will overcome these discomforts given time of one to two years, and further research should be or could be conducted after that time period in order to analyze if time has recovered discomfort and soreness. The patient (or any patient) cannot be expected to reach a full recovery in terms of comfort and previous capabilities, or even a full functional recovery within six months of time.

Measurements and MMT scale discussion

As the patient's exercises increased over the months of training, the swelling within the knee decreased significantly through physical therapy. This was directly correlated to the measurement of the knee’s circumference, as a decrease in this value would directly correlate to a decrease in swelling. MMT scale was seen to increase gradually over the months of physical therapy that the patient had undergone, and is fully indicative of a gradual recovery to full ambulation and capability to exercise normalcy in ADL. Overall, by analyzing the positive trends over time in measurements of physical therapy and rehabilitation, the expected outcome of a near full recovery is achieved and is in a direct relationship with the exercises, post-exercise modalities, and soft-tissue mobilization.

Strengths and limitations of case design

Strengths

This case highlights several strengths of a structured yet adaptable postoperative strategy. First, the rehabilitation schedule was prospectively organised into clearly defined phases, each with objective exit criteria, which promoted consistent decision-making and facilitated replication in other clinics. Second, progress was monitored with a multidimensional battery ROM, MMT strength, limb circumference, pain scales qualitatively through VRS, and the LEFS-allowing triangulation of recovery rather than reliance on a single metric. Third, advancement of knee flexion in 15° weekly increments respected biomechanical data on patellofemoral contact forces, thereby protecting the fixation while minimizing retinacular adhesions. Fourth, the treating therapist adjusted exercise selection, resistance, and volume in real time according to the patient’s daily tolerance, striking an effective balance between protection and functional challenge. Finally, functional CKC drills such as sled pushes and Spanish squats were introduced as soon as radiographic union was secure, accelerating neuromuscular retraining relevant to everyday demands and enhancing patient engagement.

Limitations

However, there are several limitations, listed below, that must be considered following this case’s approach, intervention, and report.

Single case study: All findings are extracted solely from one 56-year-old woman, with no second similar cases available for research and analysis. 

Uncontrolled rehabilitation variables: While the patient’s exercise program, soft-tissue mobilization, and other modalities like heating packs and cold packs were all monitored in the clinic, the patient’s home routine was not recorded or observed in depth. ADL, at-home modalities, nutritional profile, and sleep were not recorded with accuracy.

Reliance on subjective outcome measures: The protocol for measuring the success of rehabilitation is clinician-based or patient-reported. Such tests include MMT, LEFS, and VRS. These tests are prone to inter-rater variability and day-to-day fluctuations.

Medical literature discussion

A published series on comminuted patellar fractures in middle-aged and older adults reported the typical final knee flexion to be between 120° and 135° and radiographic union within 12-20 weeks after tension-band or screw fixation [5,8]. The present patient reached 129° of active flexion and demonstrated complete cortical bridging by week 20, falling squarely within those ranges. Likewise, her LEFS score climbed from 11 to 50/80; values between 45 and 60/80 are commonly cited at 6-12 months [6], suggesting that her functional recovery is progressing at or slightly ahead of the expected tempo. Quadriceps strength deficits of 15-30% relative to the uninvolved limb are reported during the first postoperative year; the residual asymmetry in this case (4+/5 versus 5/5) is therefore typical [9]. Finally, the staged advancement from immobilization to CKC loading parallels protocols shown to minimize patellar instability in patients through completing OKC with restrictions, such as completing the exercises at low flexion angles [7].

Improvements in range of MMT, VRS, and LEFS scores occurred in direct temporal association with each planned escalation of load and complexity, supporting the premise that a criteria-driven, phase-based approach structured by expert opinion can safely accelerate functional gains after complex patellar fracture. Objective metrics, notably the 1.76 cm reduction in limb girth and the graded rises in MMT, confirm that the soft-tissue and neuromuscular adaptations targeted by the protocol were achieved. Persistent, low-grade pain and episodic stiffness underscore biological realities: extensor-mechanism healing and retinacular remodelling continue well beyond bony union, and patients benefit from guidance during this prolonged phase. Taken together, these observations justify the recommendation for continued therapy past 20 weeks and for future studies that pair similar rehabilitation algorithms with longer follow-up and larger cohorts to refine best-practice guidelines.

## Conclusions

A five-phase, criteria-driven rehabilitation plan, progressing from early protected motion through graduated isometrics to CKC functional drills, enabled a 56-year-old woman who underwent ORIF for an open, comminuted patellar fracture to advance in five months from 40° to 129° AROM, from 2-/5 to 4+/5 quadriceps strength based on MMT, and from 11/80 to 50/80 on LEFS, while pain fell from 5/5 to 3/5 on VRS and radiographs confirmed osseous union. This trajectory shows that methodical loading and daily low-intensity range-of-motion work can restore substantial mobility, strength, and independence within six months, yet residual stiffness, strength asymmetry, and psychological hesitancy often linger, underscoring the need for continued follow-up, patient education, and cautious return to high-demand activities and RTS.
